# Efficacy of Laser Pulpotomy vs. Conventional Vital Pulpotomy in Primary Teeth: A Comparative Clinical Analysis

**DOI:** 10.3390/children12030341

**Published:** 2025-03-08

**Authors:** Jasna Simonoska, Roko Bjelica, Aleksandar Dimkov, Jasmina Simjanovska, Dragana Gabrić, Elizabeta Gjorgievska

**Affiliations:** 1 Department of Paediatric and Preventive Dentistry, Faculty of Dentistry, University Ss Cyril and Methodius, 1000 Skopje, North Macedonia; jsimonoska@stomfak.ukim.edu.mk (J.S.); adimkov@stomfak.ukim.edu.mk (A.D.); egjorgievska@stomfak.ukim.edu.mk (E.G.); 2 Department of Oral Surgery, School of Dental Medicine, University of Zagreb, 10000 Zagreb, Croatia; rbjelica@sfzg.hr; 3 Institute for Lung Diseases in Children “Kozle”, 1000 Skopje, North Macedonia; jasmina.simjanovska@gmail.com; 4 Department of Dental Medicine, University Hospital Centre Zagreb, 10000 Zagreb, Croatia

**Keywords:** pulpotomy, tooth, deciduous, lasers, mineral trioxide aggregate, calcium hydroxide

## Abstract

**Background/Objectives**: Vital pulpotomy involves the partial removal of the coronal pulp tissue in cases of vital pulp exposure, aiming to maintain the health and function of the remaining radicular pulp. The first aim of this study was to compare the effects of two different methodological approaches towards vital pulpotomy of the primary teeth—conventional and laser pulpotomy; thereafter, the aim was to perform a comparison of the effects of the application of calcium hydroxide (Ca(OH)_2_) versus mineral trioxide aggregate (MTA); then, we analyzed and compared clinical and radiographic changes in pulpotomized teeth over a defined time period; finally, we determined the degree of efficiency of different methods of vital pulpotomy of the primary teeth. **Methods**: This study analyzed 40 primary molars, 20 teeth treated with conventional pulpotomy and 20 teeth submitted to laser pulpotomy. Additionally, two pulpotomy agents—Ca(OH)_2_ and MTA—were used. The following clinical parameters were examined: pain, percussion sensitivity, swelling, sinus tract presence and pathological mobility. Absence of apical radiolucency, external and internal root resorption, and periodontal ligament widening were key radiographic parameters of the success of the endodontic therapy. **Results**: The results showed that there was no statistically significant difference between the two pulpotomy agents—Ca(OH)_2_ and MTA—and therefore both can be used as alternative pulpotomy agents in the primary teeth. **Conclusions**: The success rate of pulpotomy with Ca(OH)_2_ is similar to that of pulpotomy with MTA. The combination of laser pulpotomy and the use of MTA gave the best results according to all clinical and radiographic criteria examined and in all analyzed periods.

## 1. Introduction

Despite recent breakthroughs in dental caries prevention and the greater knowledge of the necessity of preserving natural primary dentition, the decayed, filled, and missing teeth (DMFT) index remains high, and many teeth are still lost prematurely [1]. Dental caries and dental trauma are the two most common causes of early tooth loss in children [2]. This can result in malocclusion, which can cause cosmetic, phonetic, and functional issues that can be temporary or permanent. As a result, the major goal of pulp therapy is to preserve the integrity and health of the oral tissues [3].

The major purpose of primary tooth pulp therapy—pulpotomy and pulpectomy treatments—is to keep teeth in the mouth and preserve them, preventing early extraction and allowing for the timely removal of primary dentition without needless difficulties. The intricate root canal systems in primary teeth, coupled with the close proximity of the permanent tooth germ and the difficulty in achieving ideal root canal fillings that resorb physiologically, have led to pulpotomy being established as the primary treatment modality for deciduous dentition [4,5].

Pulpotomy in primary teeth can be defined as the amputation of the affected and infected coronal portion of the pulp tissue preserving the vitality and function of the entirety or part of the remaining radicular pulp [6]. The main indications for pulpotomy of deciduous teeth, according to the American Academy of Pediatric Dentistry, are exposure of the pulp in the treatment of caries in normal, vital pulp, or in reversible pulpitis; traumatic pulp exposure; assessment of radicular pulp tissue that should be vital, with no suppuration, necrosis, or prolonged hemorrhage that cannot be stopped with a cotton swab for several minutes and no radiographic signs of an infection and/or path [7].

Materials used in vital pulp treatment (VPT) should have several characteristics, including the capacity to eradicate microorganisms, form a sufficient seal, and stimulate mineralization and normal root growth. Buckley originally developed formocresol (FC) in 1904 [8]. It has since been regarded as the “gold standard,” becoming the most extensively used medicine because of its bacteriostatic and fixative qualities in pulpotomy in primary teeth, with a high success rate [9].

This has prompted scientists to seek more suitable alternatives such as calcium hydroxide—Ca(OH)_2_—MTA, laser radiation, and others. Ca(OH)_2_ is widely known as one of the earliest used materials in pulpotomy. MTA is a viable alternative for formocresol and has been effectively employed as a pulp-coating agent in pulpotomized primary molars [9]. Mineral trioxide aggregate (MTA) was described by Torabinejad et al. in 1995 [10] as a biocompatible dentin bridge-inducing substance that was utilized as a pulpotomy agent. MTA’s demonstrated clinical effectiveness is likely attributable to its mechanism of action, which includes the formation of calcium hydroxide, calcium ion release promoting cell attachment and proliferation, and the modulation of cytokine production [11]. It promotes hard tissue-producing cell development and migration, leading to the production of hydroxyapatite on the MTA surface, which offers a biological seal [12,13].

The use of lasers is one of the more recent improvements to essential pulpotomy in deciduous teeth. Laser applications in pediatric dentistry practice might be an alternative, a supplement, or a replacement for existing treatments in a variety of soft and hard tissue treatment procedures. The abbreviation LASER stands for light amplification by stimulated emission of radiation, i.e., any device that generates and directs a narrow, concentrated beam of light with coherent photons [14]. The atoms or molecules of the laser medium—whether a ruby or crystal in the form of a gas or a liquid—are “pumped” in a laser such that the majority of them have higher energy levels than in the initial condition. Regarding the pulp tissue, laser irradiation increases the creation of calcified nodules in human dental pulp fibroblasts, as well as collagen and osteocalcin synthesis alkaline phosphatase activity. Laser treatment results in a superficial layer of coagulation necrosis, creating a barrier between the pulp and the subbase while remaining compatible with the deeper tissues [15]. The diode laser is commonly used for soft tissue surgery, periodontal pocket treatment, and peri-implantitis, but it may also be utilized in endodontics for root canal disinfection and laser-assisted tooth whitening [16,17,18]. The diode laser irradiation is significantly absorbed by the pigmented tissues, comprising hemoglobin, melanin, and collagen chromospheres, while hard dental tissues absorb less. As a result, this wavelength is suitable for cutting, vaporization, blood coagulation, curettage, hemostasis, and oral soft tissue surgery in areas around dental structures [19,20,21].

The main purpose of the present study is to determine the effectiveness of various methods of vital pulpotomy in deciduous teeth. The null hypothesis tested was that there is no difference between conventional and laser pulpotomy, analyzed by the clinical and radiographic parameters, as well as that there is no difference between Ca(OH)_2_ versus MTA as a pulpotomy agent.

## 2. Materials and Methods

### 2.1. Study Design and Inclusion Criteria

Thirty-seven patients, aged four to nine years, referred to the Department of Pediatric and Preventive Dentistry, Faculty of Dentistry, University Ss Cyril and Methodius, were chosen at random. The Institutional Ethical Committee granted ethical approval (protocol code: 02-3362/3). All parents of children participating in the study provided informed consent. There was no systemic disease in any of the individuals.

The study included a total number of 40 carious primary molars.

Inclusion criteria:

1. Clinical criteria: Absence of spontaneous pain, absence of swelling, no tenderness on percussion, no pathological mobility, absence of sinus tract, and no initially unsuccessful hemorrhage control.
2. Radiographic criteria: Teeth with no radiographic signs of inter-radicular bone loss, no loss of lamina dura and periodontal ligament widening, and no physiological root resorption of more than one-third of the root.

Exclusion criteria: primary molars with swelling or fistula, non-restorable teeth, teeth with marked tenderness to percussion, teeth with excessive mobility, teeth with radiolucency in the furcal or periapical area, teeth with spontaneous pain (especially at night), teeth with necrotic pulp, teeth close to exfoliation, parents/patients who are unwilling to participate in the research, and any medical history that would rule out pulp therapy. An equal number of patients were randomly assigned to each of the two treatment groups. Patients in the first group had conventional endodontic therapy, whereas patients in the second group received diode laser treatment. Each group was divided into two subgroups.

The first (control) group consisted of 20 primary molars, which were treated by the conventional method of vital pulpotomy using local anesthesia and a sterile bur:

First subgroup (10 teeth)—Ca(OH)_2_ applied as a pulpotomy agent;
Second subgroup (10 teeth)—MTA applied as a pulpotomy agent.

The second group consisted of 20 primary molars, which were treated by laser vital pulpotomy:

First subgroup (10 teeth)—Ca(OH)_2_ applied as a pulpotomy agent;
Second subgroup (10 teeth)—MTA applied as a pulpotomy agent.

### 2.2. Clinical Procedure

The approach was “one visit” and was carried out by the following clinical protocol:

Local anesthetic was used to manage pain effectively. Lidocaine (Lidokain Belupo^®^ 100 mg/mL spray lidocaine, Koprivnica, Croatia) was used prior to the infiltration of the anesthetic (infiltrative or block anesthesia depending on the location). As an anesthetic, Scandonest 2% with vasoconstrictor (Mepivacaine HCl. 2% Levonordefrin 1: 20,000 Injection, USP, Septodont, Saint-Maur-des-Fossés, France) was used.

#### 2.2.1. Conventional Pulpotomy

Isolation with a rubber dam or OptraGate (Ivoclar Vivadent AG, Schaan, Liechtenstein) was employed. The roof of the pulp chamber roof was removed, and the coronal pulp tissue was excavated by using a sterile sharp spoon excavator or a No. 4 or 6 slow-speed bur. Sterile cotton pellets with mild pressure were used to stop bleeding from the pulp tissue at the root canal orifices. The pulp chamber was later irrigated with saline. A sterile moist cotton pellet soaked in saline and placed over the severed root stumps for five minutes under pressure was used to reduce post-amputation hemorrhage. The severed stumps were then examined for bleeding. If the hemorrhage persisted, the tooth was disregarded from the study; if the hemorrhage was effectively controlled, the pulpotomy medicaments were applied according to the following groups.

Following the traditional pulpotomy, in the first subgroup, Ca(OH)_2_ (Calcident 450^®^, Willman & Pein GmbH, Barmstedt/Hamburg, Germany) was applied at the bottom of the pulp chamber covered with a layer of GIC Fuji IX GP^®^ (GC International AG, Luzern, Switzerland) The cavities were restored with composite, Tetric Evo Ceram Bulk Fill (Ivoclar Vivadent, Schaan, Liechtenstein).

In the second subgroup, after achieving hemostasis with a moist cotton pellet, the MTA paste, BIO MTA^®^ (PPH CERKAMED Wojciech Pawlowski 37-450 Stalowa Wola, Poland), was prepared and placed in the pulp chamber and softly condensed with a moistened cotton pellet, followed by the application of a layer of 3M™ Cavit™ (3M Deutschland GmbH, Neuss, Germany) in the first visit, allowing MTA to set completely. Thereafter, in the second visit, the cavities were sealed with glass ionomer restorative material GC Fuji IX GP ^®^ and composite resin Tetric EvoCeram Bulk Fill (Ivoclar Vivadent, Schaan, Liechtenstein).

#### 2.2.2. Laser Pulpotomy

During the vital pulpotomy with the diode laser, the patients, the dentist, and the assistant were all provided with protective eyeglasses during the laser application. The pulpotomy was conducted as follows: after haemostasis was achieved, the pulp was ablated up to the level of the canal orifice with a diode laser (LaserHF, Hager&Werken GmbH Co, Duisburg, Germany) at 975 nm, with 2 W of power, in continuous mode. The laser beam was administered for 10 s over the coronary pulp stumps until full hemostasis was achieved. The surface was irrigated with 3% H_2_O_2_ and 5.25% NaOCl. Following the laser pulpotomy, the same restorative technique as that used in the traditional approach was performed: application of Ca(OH)_2_ or MTA (in the different subgroups), followed by application of GIC and composite resin restoration.

### 2.3. Therapeutic Outcomes

The parents were advised to report any postoperative symptoms or pain. After one, three, and six months, patients were recalled for clinical and radiographic examinations. Restorations of pulpotomized primary molar teeth were evaluated under typical clinical settings in baseline and control sessions. The investigation including observations of marginal discoloration, anatomical shape, the presence of recurrent caries and marginal integrity. To verify that the pulpotomy success rate was not altered, clinically undesirable conditions were documented, and teeth with failed restorations were eliminated from the research.

The following criteria were used to define clinical success: absence of pain, no tenderness to percussion, absence of swelling and sinus tract fistula, and normal tooth mobility. Teeth were scored as radiographically successful if they showed no evidence of radicular radiolucency, internal or external resorption, or periodontal ligament space widening.

### 2.4. Statistical Analysis

The statistical analysis was performed using SPSS (version 25; IBM, Armonk, NY, USA). The one-tailed Fisher exact *p*-test was applied for statistical analysis, with the level of statistical significance set at *p* < 0.05.

## 3. Results

### 3.1. Clinical Outcomes

In the group of patients treated with the conventional vital pulpotomy method, where Ca(OH)_2_ was applied as a pulpotomy agent, pain was not registered after one or three months. In one patient (10.0%), pain was reported in the sixth month. In the conventional method with MTA as a pulpotomy agent, pain was registered in two patients (25.0%) in the first month. No pain was reported in the third and in the sixth month in all the patients. In the laser pulpotomy-treated group, pain was reported in one patient (10.0%) in the first and the third month, after the application of Ca(OH)_2_. No pain was reported in the sixth month. After using the laser method in combination with the MTA agent, pain was registered in one patient at the six-month control (11.1%), while in eight, patients no pain was registered (88.9%).

No fistula was observed in either the conventional or laser pulpotomy groups throughout the study period.

Swelling was not reported in the conventional Ca(OH)_2_ group at the first- and sixth-month recalls, but it was observed in one patient (11.1%) at the third month recall. In the laser Ca(OH)_2_ group, swelling was observed in one patient (11.1%) at the sixth month recall but not at the first and third month recalls. No swelling was observed in either group using MTA.

In the group treated with conventional pulpotomy with Ca(OH)_2_ as a pulpotomy agent, tooth mobility (luxation) was not present in any of the patients after the first month, while it was registered in one patient (10.0%) at the sixth month recall. Mobility in the group treated with laser pulpotomy using Ca(OH)_2_ as a pulpotomy agent was present in one patient (10.0%) after three months. Mobility was not registered with the conventional and laser method using MTA as an agent.

Premature exfoliation was not observed in the group treated with conventional pulpotomy, using Ca(OH)_2_ as a pulpotomy agent, after one and three months, although after the sixth month, it was present in one patient (10.0%). In the group treated with the laser method and Ca(OH)_2_, premature exfoliation was present in one patient (10.0%) after the third month. In both groups, premature exfoliation was not registered when MTA was used. A comparison of clinical parameters between conventional and laser pulpotomy using both pulpotomy agents is described in Table 1.

### 3.2. Radiological Outcomes

The radiological parameters of conventional and laser pulpotomy using both pulpotomy agents are described in Table 2.

Periodontal ligament widening was registered in the conventional pulpotomy group after six months in only one patient (10.0%) when Ca(OH)_2_ was applied, while in the MTA subgroup, enlargement of the periodontal ligament was registered after the third and the sixth month in two patients (20.0%). The laser method with Ca(OH)_2_ did not register periodontal ligament enlargement in the first month in any patient. After the third and sixth months, the enlargement of the periodontal ligament was registered in one (10.0%) patient. Periodontal ligament widening was not recorded in any of the patients during the study when using the laser method with MTA as a pulpotomy agent.

In the conventional pulpotomy group with Ca(OH)_2_ as a pulpotomy agent, presence of internal/external resorption was registered in one patient after three months and in two patients (20.0%) after the sixth month. When Ca(OH)_2_ and laser pulpotomy were performed, resorption was observed after the third month in two patients (20.0%), and in one patient after six months (11.1%). In the cavities where MTA after conventional pulpotomy was applied, resorption was found in two patients and in the laser treated group, it was present only after six months in one patient (11.1%).

Occurrence of furcal/periapical radiolucency was found in all treated groups, except for the laser pulpotomy with MTA group. After the conventional method with Ca(OH)_2_, radiolucency was noted in one patient both after three and six months, while in the laser-treated group, periapical radiolucency was observed in one patient after three months (10.0%); when MTA was applied, periapical radiolucency was registered after six months in two patients (20.0%).

### 3.3. Comparison of Different Methods

There was no statistically significant difference when comparing both methods with the two pulpotomy agents, in the tested time intervals, since the *p*-values were greater than the set level of significance (*p* > 0.05) (Table 3).

## 4. Discussion

This study aimed to evaluate the efficacy of various vital pulpotomy methods in primary teeth, specifically comparing conventional and laser pulpotomy techniques and the use of Ca(OH)_2_ and MTA as pulpotomy agents. The primary goal was to assess clinical and radiographic outcomes to determine the optimal approach for preserving the vitality of the remaining radicular pulp, while also considering the limitations and potential biases highlighted in the existing literature.

The teeth observed in the presented study were chosen based on the Fuks [21] criteria, and included teeth with a restorable carious lesion, absence of spontaneous discomfort, presence of at least two-thirds of the root length, no indication of internal or other forms of root resorption and easily controlled bleeding from severed locations [21,22,23].

This study did not demonstrate a statistically significant difference in clinical and radiographic outcomes between conventional pulpotomy and laser-assisted pulpotomy. Similarly, there were no statistically significant difference in clinical and radiographic success rates between Ca(OH)_2_ and MTA as pulpotomy agents. Both materials appeared to provide similar short-term efficacy in maintaining pulp health. This is consistent with some of the recent literature [24] suggesting comparable short-term efficacy in pulp preservation. However, it is important to acknowledge that some reviews highlight the potential long-term benefits of MTA over Ca(OH)_2_ in vital pulp therapy [25]. The absence of statistically significant differences in clinical parameters (pain, swelling, mobility, etc.) and radiographic parameters (apical radiolucency, periodontal ligament widening, etc.) across the treatment groups suggests that, at least in the tested follow-up period, all tested methods and materials had similar clinical performance. In the conventional pulpotomy group using Ca(OH)_2_, only one case with pain was reported at the six-month follow-up. For the MTA group, pain was noted in two patients in the first month, resolving thereafter. Other clinical parameters like sinus tract presence, swelling, mobility, and premature exfoliation were also minimal, with the majority of teeth showing no signs of these issues throughout the follow-up period for both conventional and laser pulpotomies. Radiographically, the incidence of internal resorption was limited and similar across all groups, with slight variations across follow-up times. Apical radiolucency and periodontal ligament widening were also observed only in a minority of cases. Previous studies reported that from 1 month to 9 months of follow-up, the number of teeth associated with internal resorption rose gradually when ferric sulfate, electrocautery, and laser were used [26]. Huth et al. [27] observed 4% failure due to internal resorption during a 24-month follow-up period. According to Kabaktchieva et al. [28], vital pulpotomy with MTA is a solid biological strategy for primary dental pulp therapy and should be used in clinical practice. MTA has good sealing ability, biocompatibility, cell adhesion, and pH 11–12 even after 72 days, which imparts antibacterial properties that work against certain facultative bacteria [29,30,31]. Regenerative materials such as freeze-dried bone, bone morphogenetic protein, osteogenic protein, and MTA were employed in pulpotomy by several researchers [32,33]. MTA is recognized as a good regeneration material, with success rates ranging from 97% to 100% [34]. However, these advantages did not manifest as superior clinical success in the current study, consistent with the findings of a recent systematic review conducted by Doiphode et al. [35].

While this study focuses on the clinical efficacy of both materials, it is important to acknowledge that MTA is generally more expensive than Ca(OH)_2_. This cost difference can be a significant factor for some patients, especially considering that primary teeth are eventually replaced by permanent teeth. However, a recent meta-analysis by Li et al. [36] found that MTA had a significantly higher success rate than Ca(OH)_2_ in teeth diagnosed with irreversible pulpitis and treated with partial pulpotomy. This suggests that MTA may be a more cost-effective option in the long term, as it can help to avoid the need for more extensive and expensive treatments later on. Ultimately, the choice of whether to use MTA or Ca(OH)_2_ should be made on a case-by-case basis, taking into account the individual patient’s needs and circumstances.

While laser treatment offers several advantages, it is essential to acknowledge that this study did not find a statistically significant difference between conventional and laser pulpotomy in terms of clinical and radiographic outcomes. This finding aligns with recent research, such as a systematic review and meta-analysis by Chandran et al. [37], which also reported no significant difference between laser and conventional pulpotomy techniques. Sun et al. [38] proposed laser treatment with a wavelength of 780–890 ηm, an energy of 2–3 J/cm^2^, and a power output of 100 mW in continuous mode for about 10 s with a separation distance of 2–4 mm between the laser and the target tissue. In this investigation, a 320–400 µm diameter fiber was utilized to deliver a diode laser with a wavelength of 975 ηm and an energy of 2 J/cm^2^ in continuous mode for 10 s. The technique’s noninvasive and non-pharmaceutical nature, efficient hemorrhage control, decontamination, and sterilization effect while preserving the radicular pulp, and faster pulpal wound healing that did not affect monocyte and endothelial cell inflammatory function or endothelial cell adhesion were all factors in the presented study’s clinical success. It is of vital importance to mention that low-level laser therapy (LLLT) is often cited for its anti-inflammatory and regenerative properties [39]. The presented results juxtapose those in the broader literature, which shows varied perspectives on pulpotomy techniques and agents [35]. Nevertheless, it is important to note that other studies have reported significant differences between the two treatment modalities. For example, a meta-analysis by Ansari et al. [40] found that laser pulpotomy had a significantly higher success rate than formocresol pulpotomy at 36 months. According to Niranjani et al. [41], teeth treated with lasers and Biodentine™ experienced discomfort and swelling after six months, whereas MTA therapy revealed no aberrant clinical or radiographic results and a 100% success rate.

Additionally, the initial investment and maintenance costs for laser equipment can be substantial. However, potential long-term cost benefits may arise from reduced chair time and the potential for minimally invasive procedures [37]. A comprehensive cost analysis would be beneficial to fully evaluate the economic implications of laser therapy in pulpotomy procedures.

Considering the limitations of this study, valuable results were obtained through standardized methodologies, precise outcome measurements and statistical analysis. The other factors that should be considered are potential variabilities regarding both clinicians and patients. This study did not explicitly investigate the potential influence of these factors, such as operator variability, individual oral hygiene practices, and socioeconomic elements, on the observed outcomes. These uncontrolled variables may have introduced bias and affected the treatment outcomes. It must also be recognized that, although this study did not present a meta-analysis and therefore minimized the potential effect of publication bias, there is a necessity to take into account this aspect when using existing systematic reviews to provide context to the results, because the majority of studies do not adequately report publication bias [5].

## 5. Conclusions

The current study shows a high success rate of vital pulpotomy in primary molars with Ca(OH)_2_ and MTA. There is no statistically significant difference between the two pulpotomy agents, and therefore they can be used as alternative pulpotomy agents in primary teeth. MTA is considered more effective than Ca(OH)_2_ when it is necessary to maintain long-term vitality of the pulp after the performed vital pulpotomy in primary teeth, regardless of the method used. Given the high cost of MTA and the likelihood that primary treated teeth will be replaced with new, permanent teeth, Ca(OH)_2_ may continue to be the material of choice. The existing data point to a need for enhanced research standards, extended longitudinal studies, and personalized treatment strategies to optimize outcomes in vital pulp therapy of primary teeth.

## Figures and Tables

**Table 1:** Occurrence of clinical events following conventional and laser pulpotomy using both pulpotomy agents.

Clinical Parameter		Conventional Pulpotomy						Laser Pulpotomy					
	Months	I		III		VI		I		III		VI	
	Presence/Absence	Pr [%]	Ab [%]	Pr [%]	Ab [%]	Pr [%]	Ab [%]	Pr [%]	Ab [%]	Pr [%]	Ab [%]	Pr [%]	Ab [%]
Pain	Ca(OH)_2_	0.0	100.0	0.0	90.0	10.0	90.0	10.0	90.0	10.0	90.0	0.0	90.0
	MTA	20.0	80.0	0.0	100.0	0.0	100.0	0.0	90.0	0.0	90.0	10.0	80.0
Sinus Tract	Ca(OH)_2_	0.0	100.0	0.0	90.0	0.0	100.0	0.0	100.0	0.0	100.0	0.0	90.0
	MTA	0.0	80.0	0.0	80.0	0.0	100.0	0.0	90.0	0.0	80.0	0.0	90.0
Swelling	Ca(OH)_2_	0.0	100.0	10.0	80.0	0.0	100.0	0.0	100.0	0.0	100.0	10.0	80.0
	MTA	0.0	80.0	0.0	80.0	0.0	100.0	0.0	90.0	0.0	80.0	0.0	90.0
Mobility	Ca(OH)_2_	0.0	100.0	00.	90.0	10.0	90.0	0.0	100.0	10.0	90.0	0.0	90.0
	MTA	0.0	80.0	0.0	80.0	0.0	100.0	0.0	90.0	0.0	80.0	0.0	90.0
Premature Exfoliation	Ca(OH)_2_	0.0	100.0	0.0	90.0	10.0	90.0	0.0	100.0	10.0	90.0	0.0	90.0
	MTA	0.0	80.0	0.0	80.0	0.0	100.0	0.0	90.0	0.0	80.0	0.0	90.0

**Table 2:** Occurrence of radiological findings following conventional and laser pulpotomy using both pulpotomy agents.

Radiological Parameter		Conventional Pulpotomy						Laser Pulpotomy					
	Months	I		III		VI		I		III		VI	
	Presence/Absence	Pr [%]	Ab [%]	Pr [%]	Ab [%]	Pr [%]	Ab [%]	Pr [%]	Ab [%]	Pr [%]	Ab [%]	Pr [%]	Ab [%]
Periodontal Ligament Widening	Ca(OH)_2_	0.0	100.0	0.0	90.0	10.0	90.0	0.0	100.0	10.0	90.0	10.0	80.0
	MTA	0.0	80.0	20.0	60.0	20.0	80.0	0.0	90.0	0.0	80.0	0.0	90.0
Internal/External Resorption	Ca(OH)_2_	0.0	100.0	10.0	80.0	20.0	80.0	0.0	100.0	20.0	80.0	10.0	80.0
	MTA	0.0	80.0	20.0	60.0	20.0	80.0	0.0	90.0	0.0	80.0	10.0	80.0
Furcal/Periapical Radiolucency	Ca(OH)_2_	0.0	100.0	10.0	80.0	10.0	90.0	0.0	100.0	10.0	90.0	0.0	90.0
	MTA	0.0	80.0	0.0	80.0	20.0	80.0	0.0	90.0	0.0	80.0	0.0	90.0

**Table 3:** Comparison of Fisher exact test values between the conventional and laser pulpotomy with both pulpotomy agents.

	Conventional Method/Ca (OH)_2_		Conventional Method/MTA		Fisher Exact Test *p*-Values
Follow-Up [Months]	Success [%]	Failure [%]	Success [%]	Failure [%]	
I	100	0	60	20	0.1830
III	70	20	60	20	0.6647
VI	70	30	80	20	0.5000
	**Laser/Ca (OH)_2_**		**Laser /MTA**		
	Success [%]	Failure [%]	Success [%]	Failure [%]	
I	90	10	100	0	0.5263
III	80	20	100	0	0.2632
VI	70	30	77.8	22.2	0.5557

Statistically significant (*p* < 0.05).

## Data Availability

The original contributions presented in the study are included in the article, further inquiries can be directed to the corresponding author.
